# Targeting Neutrophils to Prevent Malaria-Associated Acute Lung Injury/Acute Respiratory Distress Syndrome in Mice

**DOI:** 10.1371/journal.ppat.1006054

**Published:** 2016-12-07

**Authors:** Michelle K. Sercundes, Luana S. Ortolan, Daniela Debone, Paulo V. Soeiro-Pereira, Eliane Gomes, Elizabeth H. Aitken, Antonio Condino Neto, Momtchilo Russo, Maria R. D' Império Lima, José M. Alvarez, Silvia Portugal, Claudio R. F. Marinho, Sabrina Epiphanio

**Affiliations:** 1 Instituto de Medicina Tropical de São Paulo, Universidade de São Paulo, São Paulo, Brazil; 2 Departamento de Análises Clínicas e Toxicológicas, Faculdade de Ciências Farmacêuticas, Universidade de São Paulo, São Paulo, Brazil; 3 Departamento de Imunologia, Instituto de Ciências Biomédicas, Universidade de São Paulo, São Paulo, Brazil; 4 Departamento de Patologia, Universidade Federal do Maranhão, Maranhão, Brazil; 5 Center of Infectious Diseases, Parasitology, Heidelberg University Hospital, Heidelberg, Germany; 6 Departamento de Parasitologia, Instituto de Ciências Biomédicas, Universidade de São Paulo, São Paulo, Brazil; Queensland Institute of Medical Research, AUSTRALIA

## Abstract

Malaria remains one of the greatest burdens to global health, causing nearly 500,000 deaths in 2014. When manifesting in the lungs, severe malaria causes acute lung injury/acute respiratory distress syndrome (ALI/ARDS). We have previously shown that a proportion of DBA/2 mice infected with *Plasmodium berghei* ANKA (PbA) develop ALI/ARDS and that these mice recapitulate various aspects of the human syndrome, such as pulmonary edema, hemorrhaging, pleural effusion and hypoxemia. Herein, we investigated the role of neutrophils in the pathogenesis of malaria-associated ALI/ARDS. Mice developing ALI/ARDS showed greater neutrophil accumulation in the lungs compared with mice that did not develop pulmonary complications. In addition, mice with ALI/ARDS produced more neutrophil-attracting chemokines, myeloperoxidase and reactive oxygen species. We also observed that the parasites *Plasmodium falciparum* and PbA induced the formation of neutrophil extracellular traps (NETs) *ex vivo*, which were associated with inflammation and tissue injury. The depletion of neutrophils, treatment with AMD3100 (a CXCR4 antagonist), Pulmozyme (human recombinant DNase) or Sivelestat (inhibitor of neutrophil elastase) decreased the development of malaria-associated ALI/ARDS and significantly increased mouse survival. This study implicates neutrophils and NETs in the genesis of experimentally induced malaria-associated ALI/ARDS and proposes a new therapeutic approach to improve the prognosis of severe malaria.

## Author Summary

A deeper understanding of the pathogenesis of malaria-associated ALI/ARDS may help in the development of new therapeutic approaches to improve the prognosis of severe cases of malaria. Using the *Plasmodium berghei* ANKA-infection mouse model of ALI/ARDS, which resembles the human disease in many aspects, this study reports the critical role of neutrophils in the pathogenesis of this syndrome. Mice developing ALI/ARDS showed abundant lung-infiltrating neutrophils in association with the increased production of neutrophil-attracting chemokines, myeloperoxidase and reactive oxygen species. The parasites *Plasmodium falciparum* and *P*. *berghei* ANKA both induced the formation of neutrophil extracellular traps *ex vivo*. By targeting neutrophils and neutrophil extracellular traps with specific drugs, we succeeded in preventing the development of malaria-associated ALI/ARDS and significantly increased mouse survival.

## Introduction

Malaria is one of the most common infectious diseases and is an enormous public-health problem. In some individuals, a *Plasmodium* infection results in severe malaria. The main complications associated with this severe disease are cerebral malaria, metabolic acidosis, severe anemia, placental malaria, renal and hepatic insufficiency and acute lung injury/acute respiratory distress syndrome (ALI/ARDS), which can occur alone or in combination [1,2]. Patients infected with *Plasmodium falciparum*, *Plasmodium vivax*, and *Plasmodium knowlesi* can develop ALI or ARDS, with a mortality rate close to 80% [3].

Neutrophils are one of the key cells in the pathophysiology of ALI/ARDS driven by various conditions [4]. They are recruited to lung tissue where they release reactive oxygen (ROS) and nitrogen species (RNS), cationic proteins, such as myeloperoxidase (MPO); lipid mediators; inflammatory cytokines; elastase and matrix metalloproteinases. Although these molecules are toxic to invading pathogens, they also promote epithelial and endothelial damage [5–8]. The accumulation of proteinaceous substances [7,9] and the inefficient phagocytosis of apoptotic neutrophils also promote tissue injury [10]. Post-mortem examinations of the lungs of patients with malaria-associated ALI/ARDS have shown the presence of pulmonary edema, inflammatory infiltrates and accumulated inflammatory cells, including neutrophils, in the interstitial and alveolar spaces [11,12]. Moreover, a recent report showed higher neutrophil numbers in the peripheral blood of patients with severe malaria compared with numbers in uncomplicated cases in an Indian cohort [13]. Thus, evaluating the contribution of neutrophils to malaria associated-ALI/ARDS may help clarify the cellular and molecular mechanisms involved in the pathogenesis of this disease, which is essential for the development of new therapeutic approaches.

There are several mouse models of pulmonary pathologies associated with *Plasmodium* infections with a wide range of severity [14–18] that can be used to investigate the relevance of neutrophils in malaria associated-ALI/ARDS. C57BL/6 mice infected with *Plasmodium berghei* NK65 showed evidence of damage in their alveolar epithelia, mixed inflammatory infiltrates containing macrophages, neutrophils and lymphocytes, as well as hemorrhages, severe edema and, in some cases, the development of hyaline membranes [19], which were also found in DBA/2 mice infected with *P*. *berghei* ANKA (PbA) showing symptoms of ALI/ARDS [18]. However, CD8+ T lymphocytes have been demonstrated to be important in the induction of ALI/ARDS in C57BL/6 mice infected with *P*. *berghei* NK65 [19].

Here, we used a previously reported mouse model in which 30–60% of DBA/2 mice infected with PbA die as a result of ALI/ARDS symptoms between 7–12 days post-infection (dpi) [18,20]. DBA/2 mice suffering from ALI/ARDS show a loss of integrity of the alveolar-capillary barrier, including increased vascular permeability, pulmonary edema, hemorrhaging and leukocyte infiltration. Moreover, in this mouse model, we are able to identify mice that will likely die from ALI/ARDS using predictive criteria based on respiratory parameters and the extent of parasitemia [18]. Our data highlight the important potential of understanding the molecular mechanisms of malaria-associated ALI/ARDS as a way to identify new therapeutic targets.

## Results

### Infected red blood cell (iRBC) loads in the lung are associated with the development of ALI/ARDS in PbA-infected DBA/2 mice

**Fig 1:**
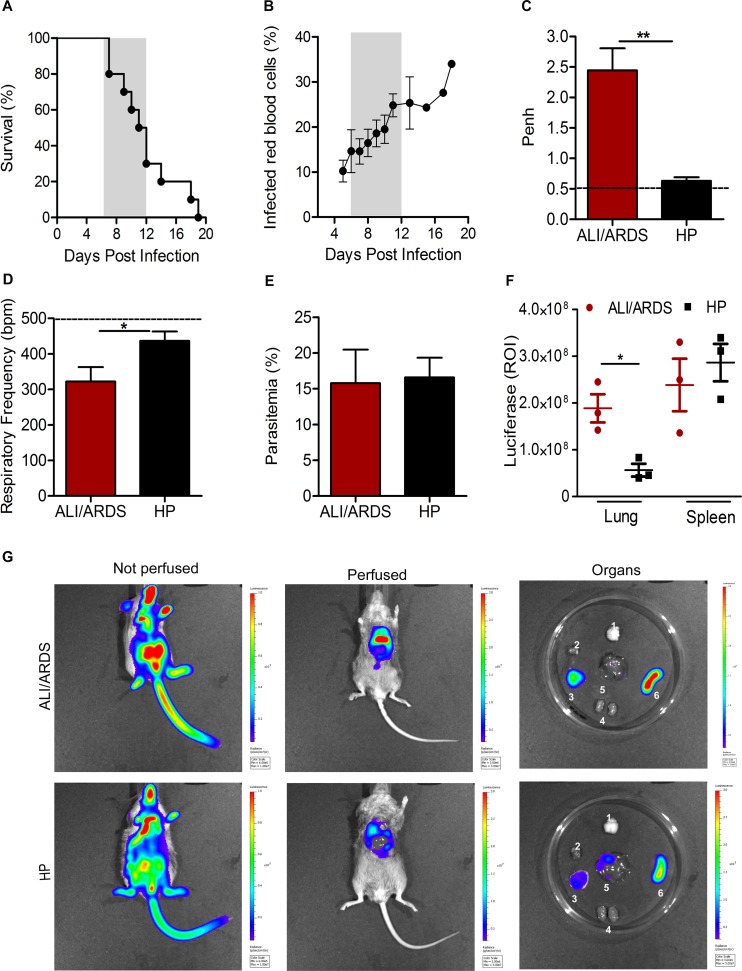
Infected red blood cells (iRBCs) loads in the lung are associated with the development of ALI/ARDS in PbA-infected DBA/2 mice. (A) Survival curve and (B) parasitemia of DBA/2 mice infected with 10^6^ *P*. *berghei* ANKA iRBCs. The gray frame shows the period between the 7^th^ and 12^th^ days post-infection (dpi) where approximately 60% of the animals died with signs of ALI/ARDS. (C) Enhanced respiratory pause (Penh) and (D) respiratory frequency (RF), as well as (E) parasitemia were measured on the 7^th^ dpi. Data are representative of five independent experiments and are expressed as the mean ± SEM (Mann-Whitney test or Kruskal-Wallis test; n = 10–12 mice/experiment; *p<0.015 and **p<0.007). (F) Bioluminescence of DBA/2 mice infected with *Plasmodium berghei* ANKA-expressing luciferase (PbA-L) on the 7^th^ day post-infection after the administration of luciferin. (T-test; n = 3 mice/group) mean ± SEM; *p <0.05). (G) ALI/ARDS-developing (hydrothorax) and HP-developing mouse (without hydrothorax) perfused or not with 1x PBS and isolated organs (1: brain; 2: heart; 3: lung; 4: kidney; 5: liver; 6: spleen). Images were captured and analyzed using an IVIS Spectrum instrument. The dashed line represents the mean value for the NI mice. NI: non-infected; ALI/ARDS: acute lung injury/acute respiratory distress syndrome; HP: hyperparasitemia.

As in our previous study [18,20], a proportion of DBA/2 mice died during the second week of PbA infection with relatively low parasitemia levels (Fig 1A and 1B). Using enhanced respiratory pause (Penh), respiratory frequency (RF) and parasitemia values as predictive criteria, we have previously shown that these mice develop ALI/ARDS and that the remaining group dies after developing hyperparasitemia (HP)[18]. Based on these parameters, mice were divided into two groups on the 7^th^ dpi (S1 Fig). The mice with ALI/ARDS showed a significantly increased Penh and decreased RF compared with the values in mice developing HP (Fig 1C and 1D), but both groups presented similar levels of parasitemia on the 7^th^ dpi (Fig 1E).

We verified parasite distribution and adhesion in various organs via bioluminescence analysis using DBA/2 animals infected with PbA expressing luciferase. Luciferin injections were made on the 7^th^ dpi, and perfused mice showed parasite loads, especially in the lungs and spleen. Moreover, mice that developed ALI/ARDS (with hydrothorax) showed more parasites in their lungs than mice developing HP (Fig 1F).

### Neutrophils are involved in the pathogenesis of malaria-associated ALI/ARDS

To investigate the mechanism responsible for the development of malaria-associated ALI/ARDS, leukocytes in the lungs and bronchoalveolar lavage fluid (BALF) were analyzed on the 7^th^ dpi.

**Fig 2:**
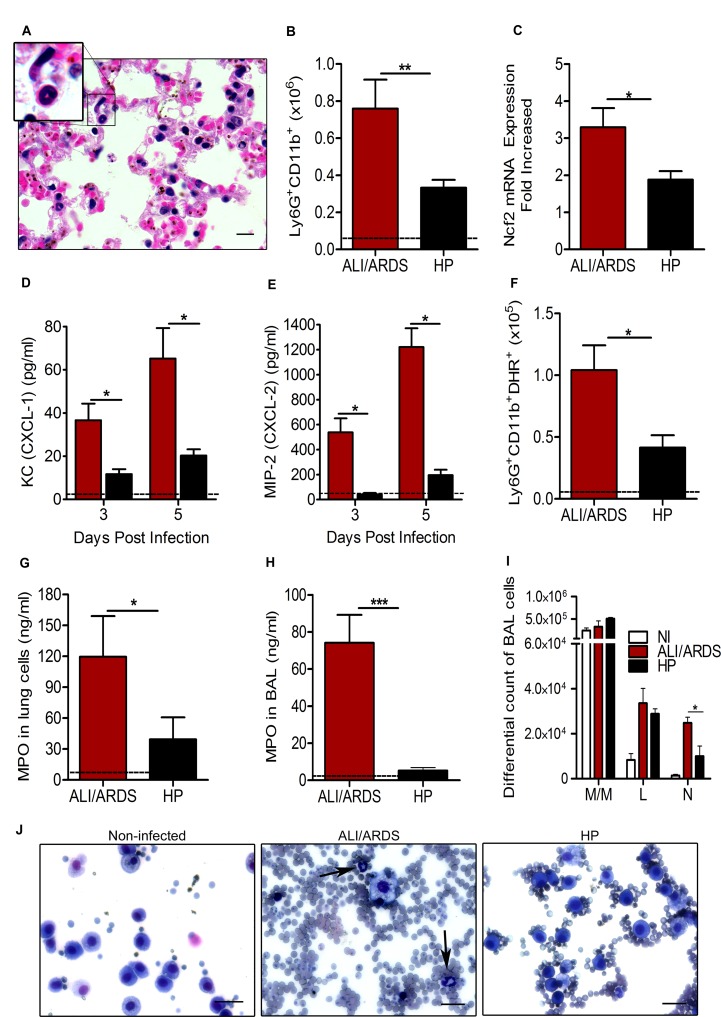
Neutrophils are involved in the pathogenesis of malaria-associated ALI/ARDS. DBA mice were infected with 10^6^ *P*. *berghei* ANKA-iRBCs. (A) Representative lung photomicrograph (630x, scale bar 10 μm) of tissue from a mouse with ALI/ARDS stained with HE, highlighting the inflammatory infiltrate, especially of neutrophils (arrows and insert) on the day of death (11^th^ day post-infection (dpi). (B) Neutrophil number (Ly6G^+^CD11b^+^) and (C) *Ncf2* gene expression in the lungs. (D) KC (CXCL-1) and (E) MIP-2 (CXCL-2) in the sera of mice with ALI/ARDS (red) and HP (black) on the 3^rd^ and 5^th^ dpi. Number of neutrophils (Ly6G^+^CD11b^+^) producing **(F)** reactive oxygen species (DHR^+^) in the lungs or producing myeloperoxidase (MPO) in the (G) lungs and (H) bronchoalveolar lavage fluid (BALF) of mice with ALI/ARDS compared with those from mice with HP. (I e J) Leukocytes in the BALF of mice. Arrowheads indicate neutrophils (400x, scale bar 25 μm). Data for three grouped experiments are expressed as the mean ± SEM. The data from figures B, C, F, G, H, I and J were collected on the 7^th^ dpi. (Mann-Whitney test and Kruskal-Wallis test; (n = 5); ALI/ARDS: n = 12–20, HP: n = 10–15; * p <0.05, ** p <0.01 and *** p <0.001). The dashed horizontal line represents the mean value for the NI mice. M/M: monocytes and macrophages, L: lymphocytes and N: neutrophils. NI: non-infected; ALI/ARDS: acute lung injury/acute respiratory distress syndrome; HP: hyperparasitemia.

Histological sections of lungs from mice with ALI/ARDS revealed an intense inflammatory infiltrate, along with numerous neutrophils (Fig 2A). Flow cytometry analysis of the inflammatory cells from the lungs showed a significantly greater increase in the Ly6G^+^CD11b^+^ neutrophil population in mice with ALI/ARDS than in those developing HP (Fig 2B). Accordingly, the mRNA expression of Ncf2, a neutrophil-specific marker, was upregulated in the lungs of mice with ALI/ARDS compared with its expression in those developing HP (Fig 2C). Consistent with neutrophil trafficking into the lungs of mice with ALI/ARDS, chemoattractants known to be produced by macrophages [21] and associated with ALI/ARDS-related injuries [22], such as the chemokines KC (CXCL-1) and MIP-2 (CXCL-2), were found at higher concentrations in sera of mice with ALI/ARDS than in that of mice with HP on both the 3^rd^ and 5^th^ dpi (Fig 2D and 2E).

Additionally, on the 7^th^ dpi, neutrophils from the lungs of mice with ALI/ARDS showed increased ROS production (DHR^+^) compared with those from mice developing HP (Fig 2F and S2 Fig). Likewise, we found increased MPO activity in the lungs (Fig 2G) and BALF (Fig 2H) of mice with ALI/ARDS on the 7^th^ dpi, indicating neutrophil activity within the alveolar space. In addition, macrophage/monocyte populations were prevalent in the BALF on the 7^th^ dpi, and they did not differ between the different groups. In contrast, the number of neutrophils was significantly higher in mice with ALI/ARDS than in those developing HP (Fig 2I), and more iRBCs were visualized in the BALF of mice with ALI/ARDS (Fig 2J). All together, these data indicate that neutrophils are recruited in higher numbers and are more active in mice with ALI/ARDS than in mice developing HP.

### Early depletion of neutrophils protects mice from ALI/ARDS

**Fig 3:**
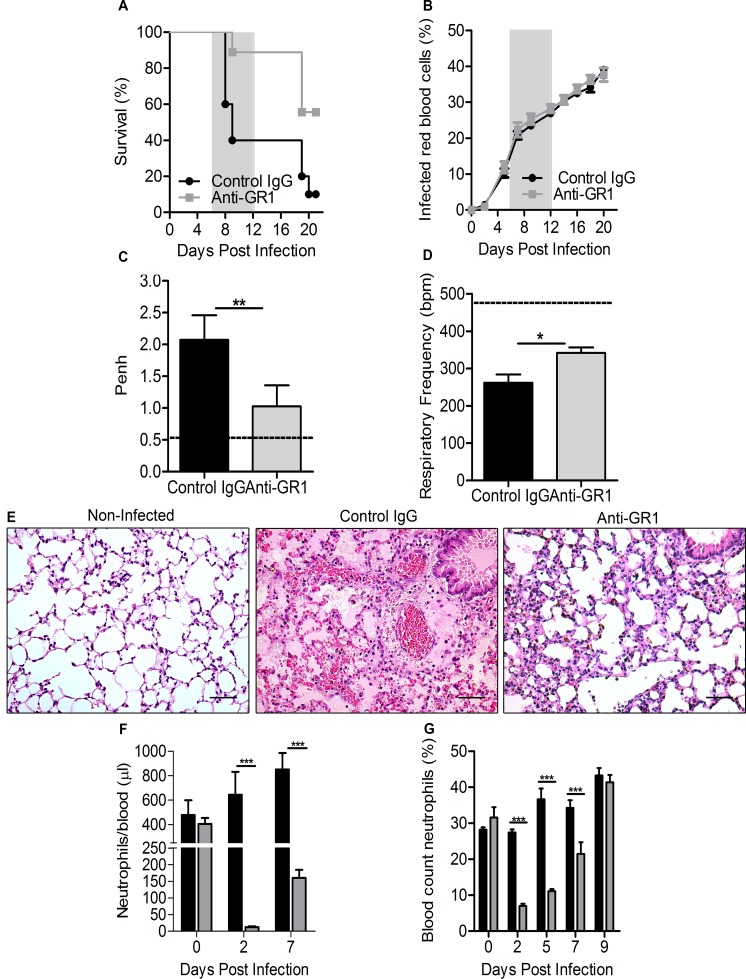
Early depletion of neutrophils protects DBA/2 mice from ALI/ARDS. (A) Survival and (B) parasitemia curves in *P*. *berghei* ANKA-infected mice treated with 0.2 mg/kg of anti-GR1 or IgG antibodies (control group) on the 1^st^ day post-infection (dpi). Respiratory parameters (C) enhanced pause (Penh) and (D) respiratory frequency on the 7^th^ dpi. (E) Photomicrographs of lung tissue on the day of death from non-infected mice, control mice (8^th^ dpi) and anti-GR1 treated mice (18^th^ dpi) (400x, scale bar 25 μm). Number/frequency of neutrophils in the blood from infected control (black) and anti-GR1-treated (gray) mice measured via (F) flow cytometry and (G) an analysis of the blood smears. Data are representative of three independent experiments and are expressed as the mean ± SEM (A, log-rank test and Wilcoxon-Gehan-Breslow test, p<0.05; Mann Whitey-test, * p <0.05, ** p <0.01 and *** p <0.001; n = 15–20 mice/experiment). The dashed line represents the mean value for the non-infected mice (NI).

To investigate the relevance of neutrophils in the development of malaria-associated ALI/ARDS, DBA/2 mice infected with PbA received a single dose of anti-GR1 IgG antibody (RB6-8C5) or an isotype control IgG (control group) on the 1^st^ dpi. Consistent with a key role of neutrophils in the establishment of ALI/ARDS, none of the anti-GR1-treated mice developed the syndrome, and their survival rate was higher than that of the control group, in which, as expected, 60% of the mice died with signs of ALI/ARDS between 7 to 12 dpi (Fig 3A). No differences in parasitemia were observed between the anti-GR1-treated mice and isotype-control-treated mice (Fig 3B). Accordingly, on the 7^th^ dpi, the anti-GR1-treated mice presented a decrease in Penh and an increase in RF compared with values in the isotype-control-treated mice that developed ALI/ARDS (Fig 3C and 3D).

Severe pulmonary edema and alveolar hemorrhaging were observed in histological lung sections of the isotype-control-treated mice, while the anti-GR1-treated mice showed only a thickening of the interstitium on the day of death (Fig 3E). The depletion of neutrophils in the anti-GR1-treated mice was effective from the 2^nd^ to 7^th^ dpi (1^st^ to 6^th^ day post-antibody treatment) (Fig 3F and 3G). However, on the 9^th^ dpi (8^th^ day post-antibody treatment), the neutrophil population in the blood returned to control levels. On the 1^st^ day post-anti-GR1 treatment, a small and transient decrease in blood monocytes was also observed, followed by an increase in monocytes on the 5^th^ dpi (S3A and S3B Fig). An increase was also observed in blood lymphocytes from the 2^th^ to 7^th^ dpi (S3C and S3D Fig), which might be a compensatory mechanism in response to the lack of neutrophils.

### Treatment with a CXCR4 antagonist protects mice from ALI/ARDS

**Fig 4:**
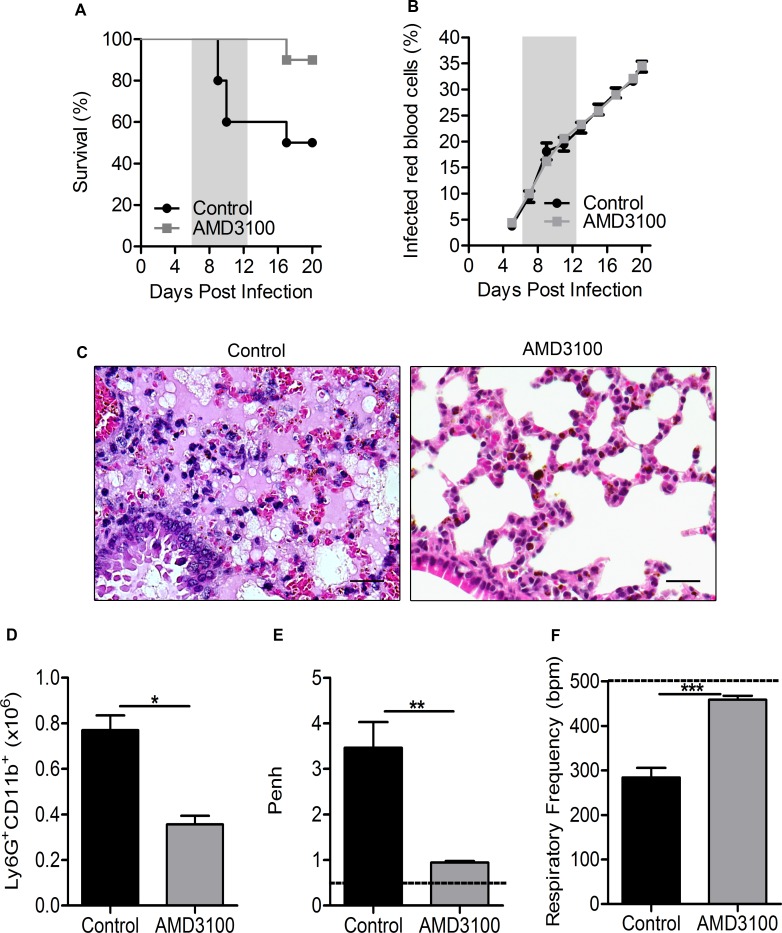
CXCR4 antagonist treatment protects DBA/2 mice from ALI/ARDS. (A) Survival and (B) parasitemia curves in *P*. *berghei* ANKA-infected mice treated with 5 mg/kg of AMD3100 or DMSO + saline solution (control group) on the 1^st^, 3^rd^, 5^th^ and 7^th^ days post-infection (dpi). (C) Lung tissues from untreated mice (CTR) (died on the 9^th^ dpi with ALI/ARDS) and AMD3100-treated mice (died on the 21^st^ dpi due to hyperparasitemia) (400x, scale bar 25 μm). (D) Number of neutrophils in the lungs, measured via flow cytometry, from infected AMD3100-treated mice and control mice (CTR). (E) Enhanced respiratory pause (Penh) and (F) respiratory frequency (RF) data for these mice. Data are representative of three independent experiments and are expressed as the mean ± SEM. The data from (D) to (F) were collected on the 7^th^ dpi. (A, log-rank test and Wilcoxon-Gehan-Breslow test, p<0.05; Mann Whitey-test, *p<0.05, ** p<0.01 and *** p<0.001; n = 10–15 mice/experiment). The dashed line represents the mean value for the non-infected mice (NI).

The CXCR4/CXCL12 signaling pathway is central to the migration of neutrophils and fibroblasts to the lung tissue in response to lung injuries due to different causes [23–26]. Hypothesizing that the CXCR4/CXCL12 axis would be important in this model of malaria-associated ALI/ARDS, we investigated its role. We used a plerixafor, AMD3100, which is a non-peptide bicyclam derivative compound that antagonizes CXCR4 and inhibits its binding to CXCL12 and thus its function [27,28], preventing neutrophil accumulation. DBA/2 mice infected with PbA were treated with four doses of AMD3100, on the 1^st^, 3^th^, 5^th^ and 7^th^ dpi. Surprisingly, between the 7^th^ and 12^th^ dpi, 60% of untreated mice died with signs of ALI/ARDS, whereas only 10% of the mice treated with AMD3100 developed the disease, and 90% survived until the end of the experiment (Fig 4A), despite there being no differences in parasitemia between the groups (Fig 4B). The untreated mice that developed ALI/ARDS presented pleural effusion, hemorrhaging and intense inflammatory infiltration on the day of death (Fig 4C); however, AMD3100 treated-mice showed only minimal thickening of the alveolar septa on the day of death, indicating that blocking the action of CXCR4 with AMD3100 was effective in reducing the pathological responses associated with the decrease in neutrophils in the lungs of AMD3100-treated mice, on the 7^th^ dpi (Fig 4D). In addition, bone marrow cellular density was higher in the AMD3100-treated mice than in the untreated mice on the day of death, indicating greater cellular retention (S4 Fig). The respiratory parameters on the 7^th^ dpi were also altered: Penh was decreased, and RF was increased in the AMD3100-treated mice compared with values in the control mice (Fig 4E and 4F).

### *Plasmodium*-induced neutrophil extracellular trap (NET) formation contributes to the pathogenesis of malaria-associated ALI/ARDS

**Fig 5:**
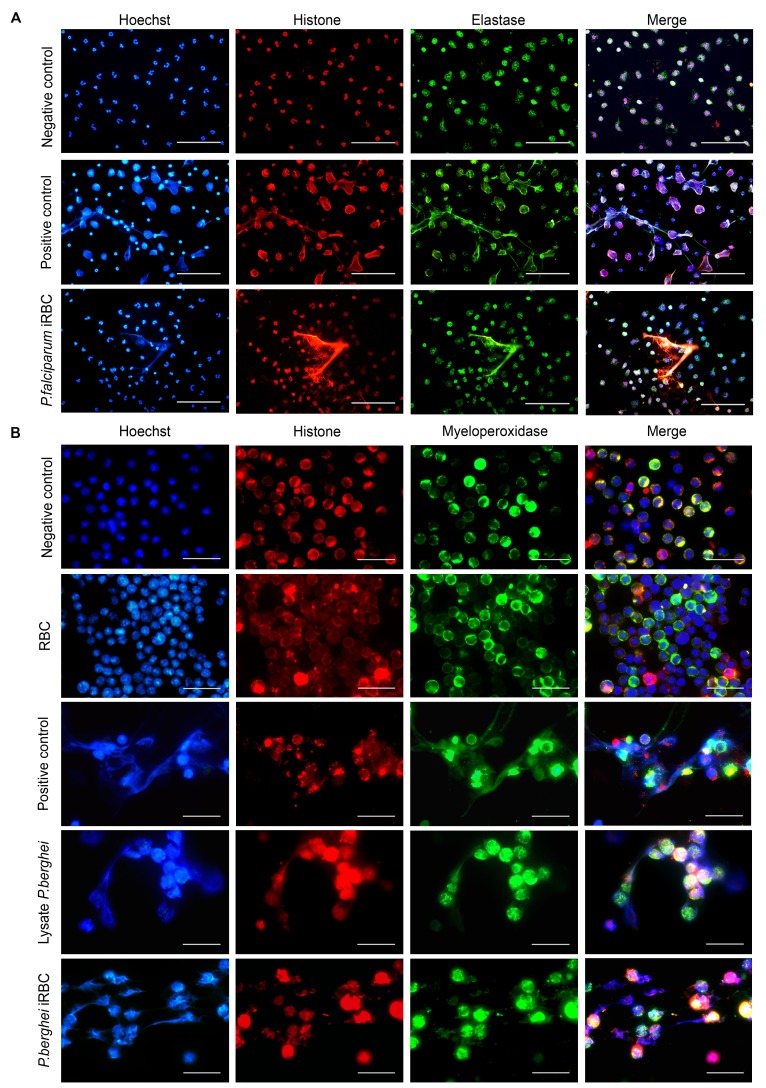
*Plasmodium falciparum* and *Plasmodium berghei* induce neutrophil extracellular trap (NET) formation. (A) Immunofluorescence of a human neutrophil culture stimulated with PMA (positive control) and *P*. *falciparum* (iRBCs) stained for neutrophil elastase (green), histone (red) and DNA (blue). Overlapping colors indicate filamentous structures (NETs) (400x, scale bar 25 μm). (B) Immunofluorescence of a DBA/2 mice neutrophil culture stimulated with PMA (positive control), red blood cells (RBCs), *P*. *berghei* lysate and *P*. *berghei* ANKA (iRBCs) stained for myeloperoxidase (green), histone (red), and DNA (blue) with overlapping pictures (1000x, scale bar 20 μm).

**Fig 6:**
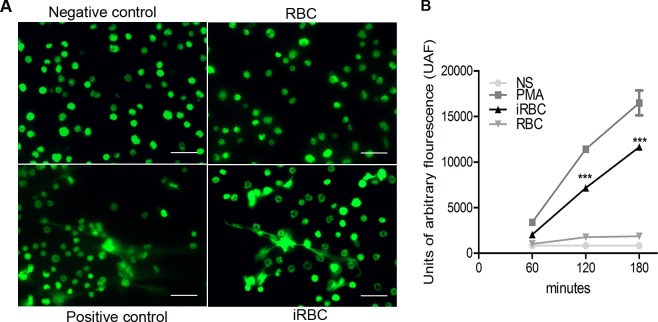
*Plasmodium berghei*-iRBCs promote more NETs than RBCs in mouse neutrophils. (A) Immunofluorescence of non-stimulated (NS) DBA/2 mouse neutrophils and of neutrophils stimulated with PMA [positive control (PC)], red blood cells (RBCs), and *P*. *berghei* ANKA (iRBCs) stained with Sytox Green (630x, scale bar 20 μm). (B) Quantification of extracellular DNA (NETs) using Sytox Green based on units of arbitrary fluorescence (UAF). Measurements were performed after 60, 120 and 180 minutes of stimulation. The data are representative of three independent experiments with mean ± SEM (Kruskal-Wallis test where *** p <0.001, between iRBC and RBC).

Our previous results show increases in neutrophil recruitment and in ROS and MPO production in the lungs of mice developing ALI/ARDS, all of which are favorable conditions for NET formation. The generation of ROS and the release of MPO are, respectively, the initial and later steps of NET generation [27,29,30]. Hypothesizing that NETs contribute to the development of malaria-associated ALI/ARDS, we investigated if *P*. *falciparum* and PbA could induce NETosis *ex vivo*. Indeed, we observed NET formation following the stimulation of human neutrophils with *P*. *falciparum*-iRBCs (Fig 5A) and mouse neutrophils with PbA-iRBCs and an iRBC lysate (Fig 5B). In addition, we quantified NETosis *ex vivo* in mouse neutrophils using Sytox Green over 60, 120, and 180 minutes (Fig 6). The neutrophils stimulated with iRBCs showed a significant increase in fluorescence intensity from 120 minutes (confirming more NETosis) when compared with RBC-stimulated neutrophils (Fig 6B).

**Fig 7:**
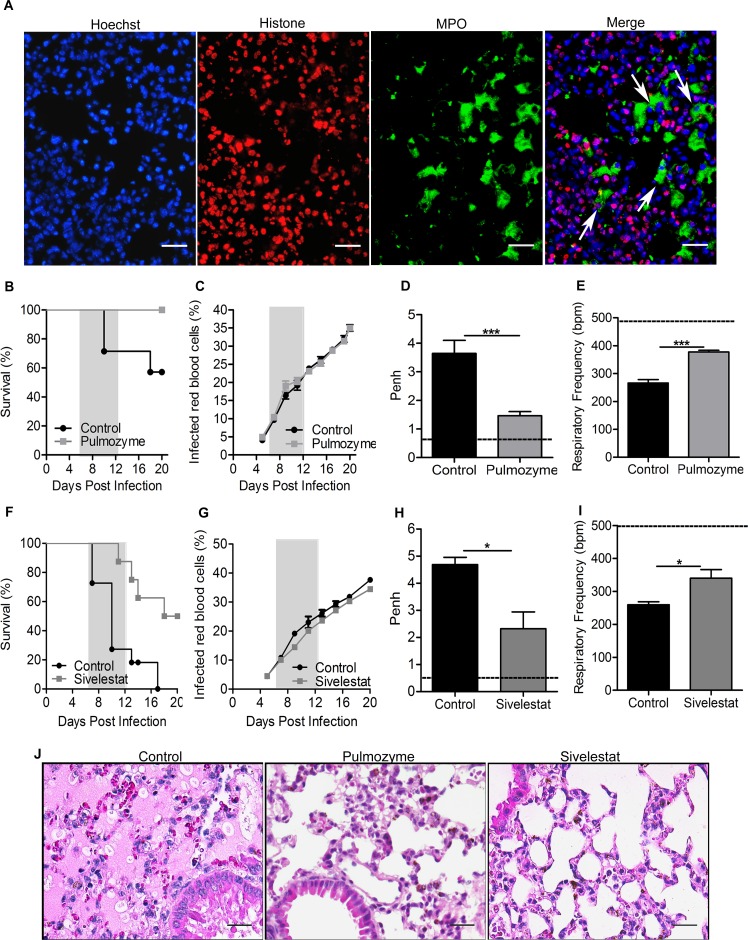
Recombinant human DNase 1 (Pulmozyme) and an elastase inhibitor (Sivelestat) improve outcomes of malaria-associated ALI/ARDS. (A) Lung sections of DBA/2 mice infected with 10^6^ *P*. *berghei* ANKA-iRBCs on the day of death (9^th^ dpi) were stained for histone (red), myeloperoxidase (MPO) (green), and DNA (blue). Overlapping colors indicate cloud-like structures. White arrowheads show selected cells forming NETs (400x, scale bar 25 μm). (B) Survival, (C) parasitemia, (D) enhanced respiratory pause (Penh) and (E) respiratory frequency (RF) on the 7^th^ days post-infection (dpi) in *P*. *berghei* ANKA-infected mice treated with Pulmozyme (50 μg/mouse) or saline solution (control group) on the 3^rd^ and 6^th^ dpi. Data are representative of three independent experiments. (F) Survival, (G) parasitemia, (H) enhanced respiratory pause (Penh) and (I) respiratory frequency (RF) on the 7^th^ dpi in *P*. *berghei* ANKA-infected mice treated with elastase inhibitor (30 mg/kg) or saline solution (control group) on the 3^rd^ and 6^th^ dpi. Data are representative of one experiment. (J) Lung tissue on the day of death from representative control mice (died on the 10^th^ dpi with ALI/ARDS), Pulmozyme-treated mice (died on the 20^th^ dpi), and Sivelestat-treated mice (died on the 20^th^ dpi) stained with HE (400x, scale bar 25 μm). Data are expressed as the mean ± SEM (C, log-rank test and Wilcoxon-Gehan-Breslow, p<0.05; Mann Whitey-test, *** p <0.001; n = 10–15 mice/experiment). The dashed line represents the mean value of non-infected mice (NI).

*In vivo*, we identified NETs in the lungs of PbA-infected mice on the day of death (Fig 7A) and also in the peripheral blood of PbA-infected mice (S5 Fig), suggesting that this mechanism is involved in malaria-associated ALI/ARDS. To ascertain the effect of NETs on the pathogenesis of ALI/ARDS, we used Pulmozyme (Roche, USA), a DNA disrupting drug, to treat DBA/2 mice on the 3^th^ and 6^th^ dpi. We observed that while 40–60% of the untreated mice died with signs of ALI/ARDS, fewer than 20% of the mice treated with Pulmozyme showed lung complications on the day of death, and none died of ALI/ARDS until the 20^th^ dpi (Fig 7B). We found no differences in parasitemia associated with or without the drug treatment (Fig 7C), but Pulmozyme-treated mice had decreased Penh and increased RF values on the 7^th^ dpi. (Fig 7D and 7E). In addition, we treated the mice with Sivelestat, an inhibitor of neutrophil elastase, and observed an increase in survival, with just 15% of mice showing pulmonary involvement (compared with the 75% of untreated mice that died of ALI/ARDS) (Fig 7F). There was no difference in parasitemia between groups (Fig 7G): however, Sivelestat-treated mice showed decreased Penh and increased RF values compared with untreated mice on the 7^th^ dpi (Fig 7H and 7I). As with Pulmozyme treated mice, elastase inhibitor-treated mice did not show histological signals of ALI/ARDS, instead they showed only minimal interstitial thickening, whereas mice in the control group died of ALI/ARDS and presented a phenotype characterized by edema, hemorrhaging, and a high influx of inflammatory cells (Fig 7J).

All together, these results show that preventing NET formation using Pulmozyme or the inhibitor elastase Sivelestat improved respiratory parameters and no signs of ALI/ARDS, such as edema, hemorrhaging, and the high influx of inflammatory cells.

### Neutrophil interactions in malaria-associated ALI/ARDS

**Fig 8:**
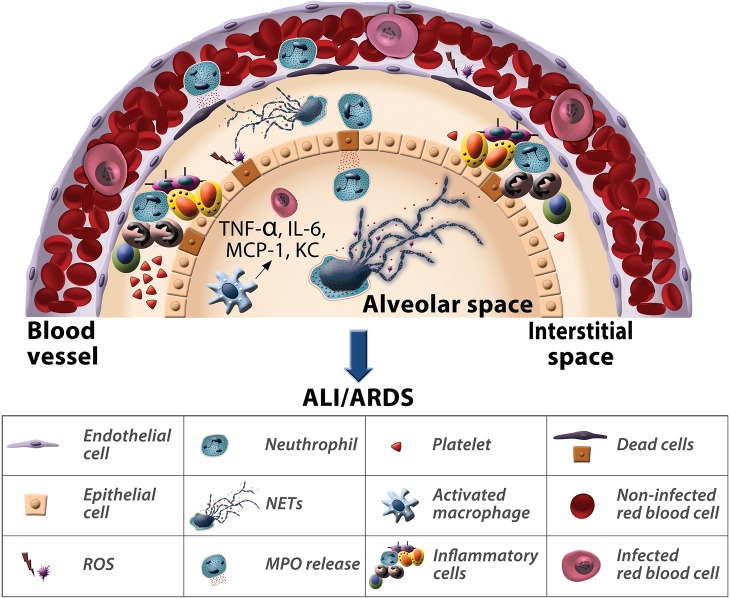
Neutrophil interactions in malaria-associated ALI/ARDS. Following *P*. *berghei* ANKA infection in DBA/2 mice, neutrophils promote the pathogenesis of ALI/ARDS. In particular, the release of myeloperoxidase and reactive species of oxygen and the formation of neutrophil extracellular traps determines the cause of death of these mice.

In Fig 8, we summarize the events leading to lung tissue damage associated with ALI/ARDS. During PbA infections, DBA/2 mice that develop ALI/ARDS show a higher number of neutrophils migrating into the lung tissue. Neutrophils from the microcirculation enter the interstitium and alveoli where they produce ROS, release enzymes (MPO) and form NETs, resulting in damage to epithelial tissue in the lungs associated with cytokine release, the death of endothelial and epithelial cells and increased vascular permeability and, consequently, edema. The course of the infection leads to hypoxemia and respiratory failure, which suggests that the use of drugs to inhibit the chemotaxis of neutrophils and the production of their inflammatory mediators may have a positive effect.

## Discussion

This study demonstrates the pivotal role of neutrophils in the establishment of malaria-associated ALI/ARDS. Using the PbA-infected DBA/2 mouse model previously established in our laboratory [18], we have shown that Penh and RF values on the 7^th^ dpi can predict whether mice will die earlier with ALI/ARDS or later with HP. Here, we show that during the development of ALI/ARDS, but not HP, neutrophils migrate and accumulate in lung inflammatory infiltrates, where they actively produce ROS and MPO, which are known to contribute to lung pathogenesis. The depletion of neutrophils, inhibition of neutrophil trafficking or inhibition of NET formation prior to the appearance of ALI/ARDS symptoms improves respiratory parameters, histopathological findings and mouse survival, thus confirming the dependency on neutrophils for establishing this syndrome.

Our data indicate that lung pathologies in this model of malaria-associated ALI/ARDS are orchestrated by neutrophils and that CXCR4/CXCL12 are the receptor/chemokine responsible for their trafficking. CXCL12 is mainly produced by bone marrow stromal cells, including vascular endothelial cells, and this receptor/chemokine complex has been associated with the trafficking of immune cells in other infectious diseases [23,30–32] and has been shown to modulate neutrophils with only minimal effects on the monocyte compartment [31]. Various diseases, especially those involving the lungs, are improved by treatments that block CXCR4 [23,33,34]. A specific CXCR4 antagonist has been shown to inhibit neutrophil accumulation into air spaces and attenuate the increase in lung permeability during LPS-induced lung injury [23]. Furthermore, it has been demonstrated that AMD310 releases neutrophils from the marginal pool in the lung into the blood but does not change the output of bone marrow neutrophils [35], as shown in our findings.

We show here that, once in the lungs, neutrophils in mice developing ALI/ARDS produce ROS and MPO, whereas those in mice developing HP do not. In other models of lung injury, it has been shown that treatment with immunoregulatory drugs decreases the production of ROS and MPO [36,37]. We hypothesize that the same effect would occur in PbA-induced ALI/ARDS, predicting that without ROS and MPO, NET formation would not occur; however, this remains to be demonstrated. In a model of ALI/ARDS associated with *E*. *coli*, it has been observed that sick animals show an increase in neutrophils in the lungs, a decrease in neutrophil apoptosis and an increase in the production of ROS and MPO by neutrophils. Moreover, animals treated with resolvin E1, a lipid mediator that reduces inflammation, showed an increase in apoptosis and a decrease in ROS, MPO, and IL-6 production by neutrophils, promoting disease resolution [38].

Previous studies showed that parasites such as *Eimeiria* spp, *Toxoplasma gondii*, *Besnoitia besnoiti*, and *Leishmania amazonensis*, among others, are able to stimulate neutrophils to form NETs [39–43], and fungal infection of *Candida albicans* hyphae and *Aspergillus fumigatus* induce NET formation in the lung of mice [44]. In addition, the presence of circulating NETs in the blood has been reported in children with uncomplicated *P*. *falciparum* malaria [45]. Here, NET formation was implicated for the first time in the development of malaria-associated ALI/ARDS. We observed NET formation *ex vivo* by stimulating human and mouse neutrophils with *P*. *falciparum* and PbA iRBCs, respectively. Most importantly, we showed that the inhibition of NET formation by treating mice with recombinant human DNase (rhDNase, Pulmozyme) or elastase inhibitor (Sivelestat) greatly reduced lung pathologies and increased mouse survival. Accordingly, it has been shown that rhDNase treatment prevents NET formation in the alveoli and, as a consequence, improves pulmonary function and diminishes hypoxia in a transfusion-related model of acute lung injury [29]. It also decreases neutrophil accumulation and activation in cystic fibrosis patients [27]. The neutrophil elastase is a serine protease present in azurophilic granules that contributes to tissue injury when released during the inflammatory process [46,47]. However, elastase inhibitor treatment in patients with SIRS has been shown to improve the symptoms of this disease, decreasing ventilation times without adverse effects during and after treatment [8,46,48].

For more than 10 years, drugs such as Pulmozyme, Sivelestat, and AMD3100 have been used in patients as adjuvant treatments of various diseases [8,46,48–52], and they are commercially available. However, the main criticism of studies conducted in Japan with elastase inhibitors is that they did not include patients with severe lung disease such as ALI/ARDS [53,54].

Therefore, our data indicate that Pulmozyme, AMD3100 and elastase inhibitors could be considered as adjuvant treatments to prevent malaria-associated ALI/ARDS in patients.

High serum levels of KC and MIP-2, selective chemoattractants for neutrophils, lead to the accumulation of these cells in the lungs. Moreover, various inflammatory factors, such as C5a, PAF, IL-8 and TNF-α, induce neutrophil activation [55]. However, it remains unclear what promotes the activation of neutrophils in the lungs of mice developing ALI/ARDS and not in those of mice developing HP. It is possible that the sequestration of iRBCs in the lungs promotes the release of different immune mediators from lung endothelial and epithelial cells. One such cytokine, IL-33, has recently been associated with pulmonary edema in severe malaria cases in Southeast Asian patients and was found to be positively correlated with neutrophils and activated monocytes [56]. It will be of interest to use the mouse model of malaria-associated ALI/ARDS to investigate if IL-33 indeed has a role in PbA-induced lung pathologies and to examine what the molecular and immune mechanisms underlying the changes may be and how they can be prevented or controlled. Studies investigating allergies and auto-immunity in the context of lung mucosa [56,57] are likely to be informative in showing how *Plasmodium* infections can develop into lung pathologies and in helping to clarifying the host-pathogen interactions that mediate these changes.

Our experimental model offers a unique view of the regulation and plasticity of the immune system in response to PbA infections, with an emphasis on lung pathology that may help understanding malaria-associated ALI/ARDS and contribute to early diagnoses and effective treatments to prevent ICU admissions and death due to ALI/ARDS, which are not yet attainable [9,18].

## Materials and Methods

### Experimental outline

Ten to twelve DBA/2 mice per group (survival group and euthanatized groups) were infected with 10^6^ *Plasmodium berghei* ANKA (PbA)-infected red blood cells (iRBCs) at the same time. In the survival group, mice showing pleural effusion or red and congested lungs at necropsy, the cause of death was designated as ALI/ARDS. For mice without pleural effusion that died after 13 days post-infection (dpi) with pale lungs and high levels of parasitemia, the cause of death was designated as hyperparasitemia (HP). By using respiratory patterns (enhanced pause and respiratory frequency) and the degree of parasitemia as predictive criteria, we established cut-off values using receiver operating characteristic (ROC) curves for these parameters measured on the 7^th^ dpi based on data from mice whose cause of death was known (survival group). Afterwards, we retrospectively diagnosed the euthanized mice as suffering from ALI/ARDS or HP by comparing their respiratory patterns and parasitemia measured on the 7^th^ dpi with the cut-off values from the survival group at the end of each experiment (20^th^ dpi), as previously described [18] and as illustrated in S1 Fig. Samples (lung tissue, blood and bronchoalveolar lavage fluid (BALF)) were collected from mice on the 7^th^ dpi from the euthanized group.

To measure chemokines, samples were collected on the 3^rd^ and 5^th^ dpi from the survival group, and lung tissues for histological analyses used to verify the cause of death were collected on the day of death.

### Ethics statement

All experiments were performed in accordance with the ethical guidelines for experiments with mice, and the protocols were approved by the Animal Health Committee of the Biomedical Sciences Institute of the University of São Paulo (CEUA n° 003, page 98, book 2), of the Tropical Medicine Institute of the University of São Paulo (n° CPE-IMT 2011/123) and of the Federal University of São Paulo (CEP 1712/09). The guidelines for animal use and care were based on the standards established by The Brazilian College of Animal Experimentation (COBEA). Ethical approval for obtaining blood from healthy adult human volunteers was provided by the Committee for Research of the University of São Paulo (Plataforma Brasil, CAAE: 11150612500005467). All the study participants gave written informed consent.

### Mice, parasites and infections

Male DBA/2 mice between 6–10 weeks old (purchased from the Department of Parasitology, University of São Paulo, Brazil) were infected with 1x10^6^ PbA (clone 1.49 L)-iRBCs, as previously described [18]. Parasitemia and mortality were monitored daily. Parasitemia was determined via Giemsa staining and expressed as the percentage of iRBCs. Mice were euthanized using ketamine (150 mg/kg)/xylazine (15 mg/kg).

### Determination of respiratory patterns

Respiratory patterns (respiratory frequency [RF] and enhanced pause [Penh]) were monitored on the 7^th^ dpi using an unrestrained whole-body plethysmography chamber (WBP, Buxco Electronics, USA) for 10 minutes (basal level), as previously described [18].

### Parasite localization via bioluminescence

DBA/2 mice were infected with PbA expressing luciferase. On the 7^th^ dpi, mice were injected with luciferin (VivoGlo Luciferin, In Vivo Grid, Catalog #: P1041, Promega), which result in the parasites becoming luminescent. Parasite localization was analyzed via an IVIS Spectrum instrument (PerkinElmer). Mice were sedated with isoflurane to take pictures (approximately 6 minutes after the injection of luciferin). Later, they were euthanized and perfused with 20 ml of PBS 1X. After perfusion, new images of the mice were captured, and their organs were then collected and placed in sterile petri dishes for observations of the bioluminescence of each tissue.

### Histopathological analyses

Lung tissues from the day of death were fixed with 10% buffered formalin for 24 hours and kept in 70% ethanol until embedding in paraffin, and sections (4–5 μm) were stained with hematoxylin-eosin (HE).

### Quantification of gene expression

Quantitative RT-PCR was performed for the relative quantification of gene expression in the lungs of non-infected and infected mice on the 7^th^ dpi. RNA extraction was performed according to the "Animal Cell I" protocol from the RNeasy Mini kit (Qiagen, USA). cDNA synthesis was performed with a 1 μg RNA sample using the First Strand cDNA Synthesis RT-PCR kit (Roche, USA) according to manufacturer's instructions. Finally, for gene expression, SYBR Green PCR Master Mix (Applied Biosystems, USA) and the 2^(-ΔΔCT)^ relative quantification method were used as described previously [58]. The qRT-PCR reactions were performed in a 7500 Fast system (Applied Biosystems, USA) with the following oligonucleotides: Ncf2 (forward—5’ gcagtggcctacttccagag 3’; reverse- cttcatgttggttgccaatg) and HPRT (forward—5’ aagcttgctggtgaaaagga 3’; reverse- 5’ ttgcgctcatcttaggcttt 3’).

### Leukocyte quantification

Lungs were collected on the 7^th^ dpi, washed with PBS 1X, and the extracellular matrix was removed with collagenase IV (Sigma-Aldrich, USA) as previously described [59]. Lung inflammatory cells were stained with fluorochrome-conjugated monoclonal antibodies (BD Pharmingen or eBioscience, USA) to the following surface molecules: CD3 (145-2C11), CD19 (1D3), Ly6G (1A8), F4/80 (BM8), CD11c (N418), CD11b (MI/70), and Ly6C (AL-21). Cells were then incubated for 30 min at 4°C (1 μg Ac/1x10^6^ cells). Data collection was performed using a FACSCantoII cytometer (BD Bioscience, USA), and analyses were performed with FlowJo VX-10 software. Blood leukocytes were measured using the same methods described above and/or in fixed blood smears stained with Instant-Prov (Newprov, Brazil).

### Reactive oxygen species assay

Lung inflammatory cells (1x10^6^) from the 7^th^ dpi were stained with antibodies for Ly6G (1A8), CD11b (MI/70), and Ly6C (AL-21) (BD Pharmingen, USA). Intracellular levels of ROS were detected in neutrophils using dihydrorhodamine 123 [(DHR123 - (1 μg/ml)]. Cells were incubated sequentially with the antibodies and DHR123 at 37°C for 30 minutes. Data collection was conducted using a FACScantoII cytometer (BD Bioscience, USA) and analyzed with FlowJo VX-10 software.

### Myeloperoxidase activity

BALF and lungs were collected on the 7^th^ dpi and sonicated for 60 seconds at 40 Hz with a *TissueRuptor* (Biospec Products Inc, USA), and the supernatant was collected. To measure myeloperoxidase levels, 100 μl of the supernatant and the substrate solution were used. The substrate solution contained citrate buffer (10 mM citric acid + 10 mM sodium citrate), 5 mg of o-phenylenediamine dihydrochloride (OPD; Sigma-Aldrich, USA) and 5 μl of H_2_O_2_ (8,8 mM). A standard curve was established with 100 μl of type II horseradish peroxidase (Sigma-Aldrich, USA) at 500 ng/ml + 100 μl of the substrate. The stop solution was made with 4N H_2_SO_4_. Sample reading was performed with a spectrophotometer (Epoch-Bio Tek, USA) at a wavelength of 492 nm.

### Chemokine quantification

The quantities of keratinocyte-derived chemokine (KC/CXCL1) and macrophage inflammatory protein 2 (MIP-2) were determined in the serum using an ELISA kit (Sigma-Aldrich, USA) according to the manufacturer's protocols, on the 3^rd^ and 5^th^ dpi among the survival group, for which the cause of death was known.

### Bronchoalveolar lavage

For BALF collection, the trachea was cannulated on the 7^th^ dpi, and lungs were washed once with 1.0 ml of PBS 1X. Total and differential cell counts from the BALF were determined from slides prepared in a cytospin and stained with Instant-Prov (Newprov, Brazil).

### Neutrophil depletion assays

The control group received IgG1 antibodies (0.2 mg/mouse), and the anti-GR1 group received anti-GR1 antibodies (RB6-8C5 isotype IgG1) (0.2 mg/mouse) on the 1^st^ dpi. Mouse blood was collected from the submandibular vein for leukocyte quantification using blood smears and flow cytometry. The ALI/ARDS phenotype was confirmed via necropsies.

### AMD3100 treatment

The AMD group received 5 mg/kg of AMD3100 in a DMSO solution (20% DMSO in 80% saline solution), and the control group received just the DMSO solution on the 1^st^, 3^rd^, 5^th^ and 7^th^ dpi. Respiratory parameters of the mice were evaluated on the 7^th^ dpi. The ALI/ARDS phenotype was confirmed via necropsies.

### *Plasmodium* synchronization and the enrichment of parasitized erythrocytes

Cultures of the *P*. *falciparum* clone 3D7 were grown as described previously [60], except that human serum was replaced with Albumax I (0.5%; Thermo, USA). Parasite multiplication was monitored via microscopic evaluations of Giemsa-stained thick blood smears. Schizont stages were purified using magnetic columns (Magnetically Activated Cell Sorting MACS Separation Columns; Miltenyi Biotec, USA) [61]. Column stabilization, washing, and elution all were carried out at room temperature with RPMI 1640 (Sigma-Aldrich, USA). To obtain mature PbA, iRBCs were synchronized as described previously [60]. Briefly, iRBCs were collected from infected mice exhibiting 10 to 20% parasitemia through a cardiac puncture and transferred to RPMI 1640 culture medium (Gibco-Thermo, USA) supplemented with 25% fetal bovine serum (FBS). The iRBCs were subsequently maintained *in vitro* at 37°C for 14 h in an atmosphere containing 5% CO_2_, 85% N_2_, and 10% O_2_. The parasitized erythrocytes were then enriched using a magnetic separation column (Miltenyi Biotec, USA) to generate cell populations consisting of approximately 95% iRBCs, as assessed based on thick blood smears. *P*. *berghei* extracts were obtained from iRBCs subjected to several freeze-thaw cycles.

### Isolation of polymorphonuclear cells from the bone marrow of DBA/2 mice

The femur and tibia of DBA/2 mice were removed, all muscles and tendons were dissected away, and the bones were placed in PBS 1X with 2% FBS and kept on ice. Bone marrow from each bone was washed with (PBS+2% FBS) and filtered through a 0.70-μm cell strainer. Cells in suspension were centrifuged at 500 x *g* for 5 minutes at 4°C, and the pellet was suspended in ice-cold distilled water for 30 seconds to lyse the erythrocytes. Then, a solution of PBS+2% FBS was used to stop the action of the distilled water, and the cells were centrifuged again. The pellet was resuspended in 1 ml PBS+2% FBS and placed on the surface of a Ficoll gradient (Sigma-Aldrich Ficoll -1119 and 1077). The samples were then centrifuged at 500 x *g* for 30 minutes at 4°C (acceleration = 5 and deceleration = 0). Neutrophils were recovered from the Ficoll ring between 1119 and 1077 and then washed with PBS+2% FBS. Finally, the neutrophils were centrifuged again, resuspended in 1 ml PBS+2% FBS, counted, and checked for purity based on Giemsa staining.

### *Ex vivo* neutrophil extracellular trap (NET) generation

Human neutrophils were obtained from peripheral blood via Ficoll gradient centrifugation followed by RBC lysis. These cells were stimulated with synchronized *P*. *falciparum*-iRBCs and phorbol-12-myristate-13-acetate (PMA, 90 nM; Sigma-Aldrich, USA) for 4 hours at 37°C in a humidified atmosphere containing 5% CO_2_. The cells were washed with PBS 1X, fixed with 4% paraformaldehyde (PFA; Sigma-Aldrich, USA) and then permeabilized with 1% Triton X-100 (Sigma-Aldrich, USA). Afterwards, we performed the NET labeling protocol.

Neutrophils isolated from the bone marrow of femurs and tibias from naïve DBA/2 mice were resuspended in PBS+2% FBS at a final concentration of 2x10^5^ cells per well. Neutrophils were stimulated with synchronized *P*. *berghei-*iRBCs, *P*. *berghei* lysate, non-infected red blood cells (RBCs) and PMA (50 nM; Sigma-Aldrich, USA) for 3 hours at 37°C in a humidified atmosphere containing 5% CO_2_. The wells were washed twice with PBS 1X, fixed with PFA (Sigma-Aldrich, USA) and permeabilized with 0.1% Triton X-100 (Sigma-Aldrich, USA).

### Analysis of NETs via immunofluorescence

To stain NETs in human neutrophils, they were incubated with rabbit anti-human-neutrophil elastase antibodies (1:1000; Santa Cruz, USA,) and mouse anti-human-H2A/H2B antibodies (1:1000; Max Planck Institute, Germany). The secondary antibodies used were donkey anti-rabbit-IgG Alexa 488 (1:200; Invitrogen, USA) and donkey anti-mouse-IgG Cy3 (1:100; Invitrogen, USA). Nuclei were stained with Hoechst 33342 (100 ng/mL; Invitrogen, USA,).

To stain NETs from DBA/2 mice, neutrophils were incubated with histone H3 goat polyclonal IgG (C-16) antibodies (1:200; Santa Cruz, USA) and myeloperoxidase polyclonal rabbit anti-human antibodies (1:400; DAKO, Denmark). Nuclei were stained with Hoechst-33342 (100 ng/mL; Invitrogen, USA). The secondary antibodies used were Alexa Fluor 488 donkey anti-rabbit and Alexa Fluor 568 donkey anti-goat (1:200; Invitrogen, USA).

To stain NETs in DBA/2 mice peripheral blood from, thin smears were made on glass slides and fixed with methanol. Immunofluorescence staining was performed using the antibodies described above_ anti-histone H3 (1:250), anti-myeloperoxidase (1:500), and the secondary antibodies (1:500). Nuclei were stained with Hoechst-33342.

Lungs from mice with ALI/ARDS or HP were collected on the 7^th^ dpi and fixed with 10% buffered formalin for 24 hours and then transferred to 70% ethanol. Tissues were embedded in paraffin, cut into sections (4–5 μm), and after deparaffinization, antigen retrieval and immunofluorescence were assessed as described previously [62] using the same antibodies as described from the *ex vivo* protocol.

### Quantification of NETs using Sytox Green

To confirm the ability of the PbA to promote NETosis, Sytox Green (Invitrogen) imaging was performed. Sytox Green is a fluorescent dye that binds to the nucleic acids of dead cells. Approximately 5 x 10^5^ mouse neutrophils (from the bone marrow) were distributed into 96-well black flat plates (Corning). Neutrophils were incubated with iRBCs or non-infected RBCs for 60, 120 and 180 minutes. As a negative control, we used Hank’s buffered salt solution (Thermo), and the positive control was established with 50 nM PMA (Sigma-Aldrich). After incubation, the supernatant was removed, and 5 mM Sytox Green (50 mL) was added. After 10 minutes, plate readings were taken at 488–523 nm in a SpectraMAX fluorimeter (Molecular Devices).

### Mice and anti-NET drug DNase

Mice were anesthetized briefly with approximately 4% halothane and 96% oxygen and then administered 50 μg/mouse (5 mg/kg) of DNAse 1 (Pulmozyme, Roche, USA) or a saline solution via intranasal spray on the 3^rd^ and 6^th^ dpi as describe previously [63]. Respiratory parameters of the mice were evaluated on the 7^th^ dpi.

### Elastase inhibitor treatment

Sivelestat (Sigma-Aldrich), an elastase inhibitor, was administered to mice (30 mg/kg) diluted in a saline solution, and the control group received just the saline solution on the 3^rd^ and 6^th^ dpi. Respiratory parameters of the mice were evaluated on the 7^th^ dpi.

### Statistical analysis

Statistical analyses and graphing were performed using GraphPad Prism 5.0 software. The data were analyzed for normality using Kolmogorov-Smirnov or Shapiro-Wilk normality tests, and variance was assessed using Bartlett’s test. Non-parametric variables were compared using Mann-Whitney tests between the groups with ALI/ARDS and HP. For the analysis of three groups, we used a Kruskal-Wallis test followed by Dunn’s post-hoc tests. For the survival curves, log-rank and Wilcoxon-Gehan-Breslow tests were used. The differences between the groups were considered significant at p≤0.05 (5%). To establish cut-off values from the data, ROC curves were generated in MedCalc version 8.2.1.0 using the results from the infected control group (survival group).

## Supporting Information

S1 Fig The sequence of events to identify mice with ALI/ARDS before death (in a euthanized group) using the parameters Penh, respiratory frequency and parasitemia as predictive criteria. (TIF)

S2 Fig Flow cytometry analysis of pulmonary cells from non-infected and *P*. *berghei* ANKA-infected mice (ALI/ARDS or HP) stained for reactive oxygen species with DHR123. (A) No doublet cells gated, (B) selected cells gated by size (SSC) and granularity (FSC), (C) Ly6G^+^ (neutrophils) gated cells and (D) dot plot for positive double-stained (Ly6G and DHR123) cells.
(TIF)

S3 Fig Monocyte and lymphocyte levels after neutrophil depletion in DBA/2 mice infected with *P*. *berghei* ANKA. (A) Number of monocytes measured via flow cytometry and (B) blood smears from infected control (CTR) and anti-GR1-treated mice. (C) Number of lymphocytes measured via flow cytometry and (D) blood smears from control (CTR) and anti-GR1-treated mice. Data are representative of two independent experiments and are expressed as the mean ± SEM (Kruskal-Wallis test where * p <0.05, ** p <0.01 and *** p <0.001; n = 10–20 mice/experiment).
(TIF)

S4 Fig DBA/2 mice are protected from malaria-associated ALI/ARDS by blocking the CXCR4 receptor. Bone marrow samples from non-infected mice, mice infected with *P*. *berghei* ANKA on the day of death (9^th^ for ALI/ARDS) and infected and treated with AMD3100 mice (21^st^ dpi). Note that the cellularity in mice infected and then treated is considerably higher than in infected, untreated mice (200x, scale bar 50 μm).
(TIF)

S5 Fig Neutrophil extracellular trap (NET) structures are present in the peripheral blood of mice infected with PbA. **Smear** of peripheral blood of PbA infected DBA2 mice stained for DNA (blue), histone (red), and myleporoxidase (green). Overlapping colors indicate filamentous structures (NETs). (1000x, scale bar 20 μm).
(TIF)
