# Correction: Targeting Neutrophils to Prevent Malaria-Associated Acute Lung Injury/Acute Respiratory Distress Syndrome in Mice

**DOI:** 10.1371/journal.ppat.1006730

**Published:** 2017-11-15

**Authors:** Michelle K. Sercundes, Luana S. Ortolan, Daniela Debone, Paulo V. Soeiro-Pereira, Eliane Gomes, Elizabeth H. Aitken, Antonio Condino-Neto, Momtchilo Russo, Maria R. D' Império Lima, José M. Alvarez, Silvia Portugal, Claudio R. F. Marinho, Sabrina Epiphanio

There is an error in the 7th author’s name. The correct name should be Antonio Condino-Neto. As a result, the name is indexed incorrectly. The name should be indexed as Condino-Neto A.
