# Supplementary figures and images for: Targeting Neutrophils to Prevent Malaria-Associated Acute Lung Injury/Acute Respiratory Distress Syndrome in Mice
Source: PLoS Pathog. 2016 Dec 7;12(12):e1006054. doi: 10.1371/journal.ppat.1006054 (PMC5142790; doi:10.1371/journal.ppat.1006054)

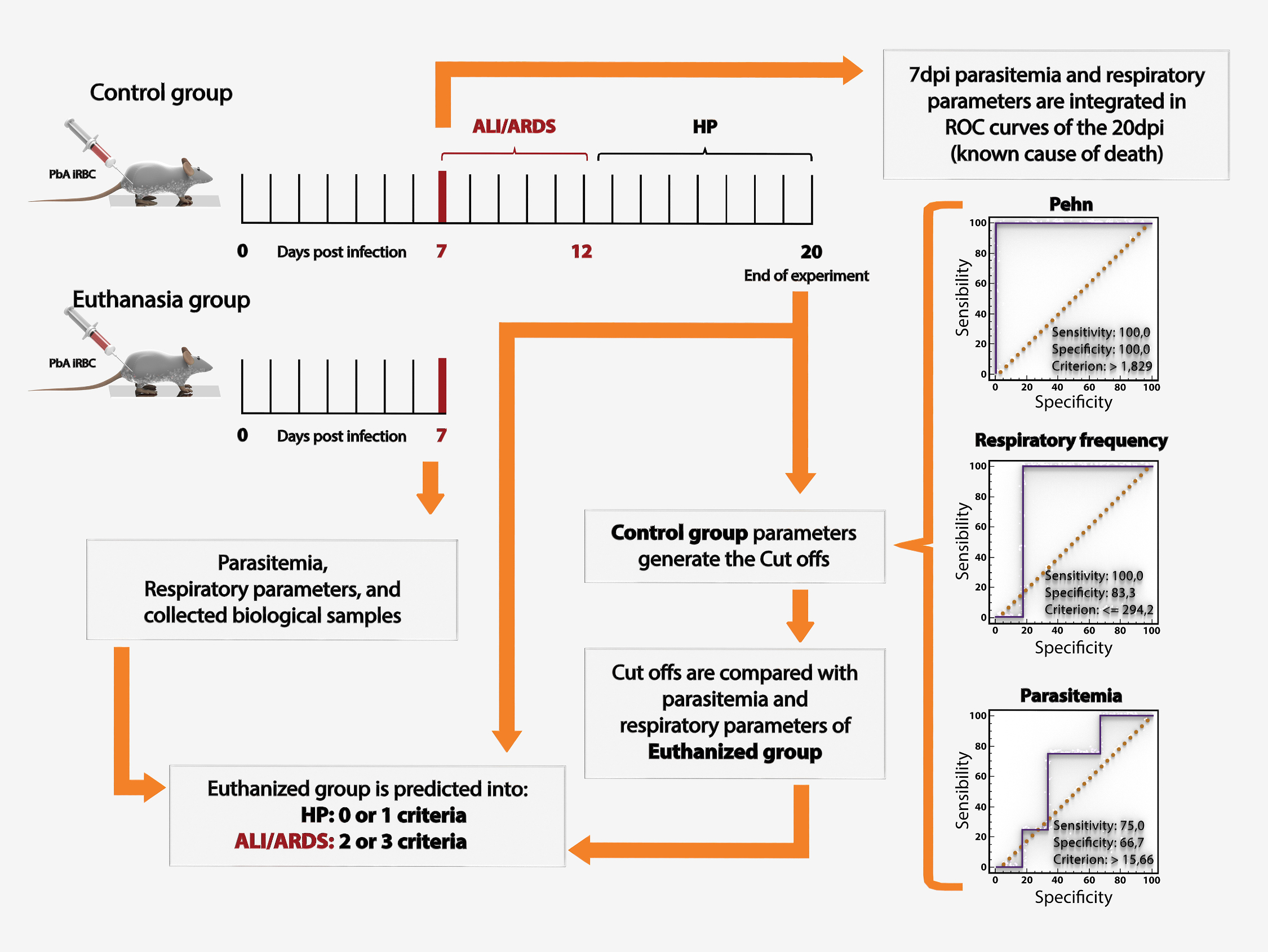

Supplement: S1 Fig — (TIF) [file ppat.1006054.s001.tif]

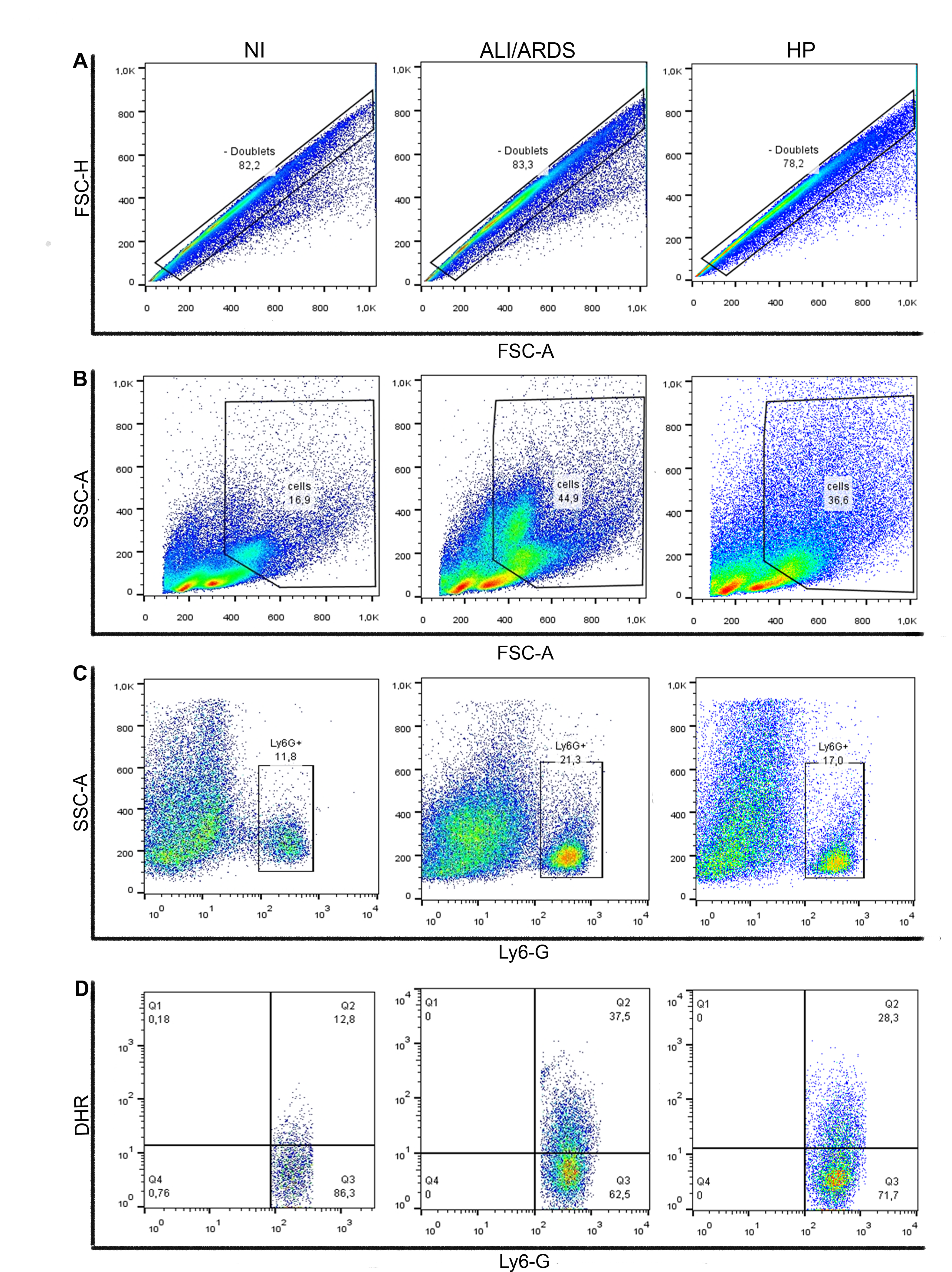

Supplement: S2 Fig — (A) No doublet cells gated, (B) selected cells gated by size (SSC) and granularity (FSC), (C) Ly6G+ (neutrophils) gated cells and (D) dot plot for positive double-stained (Ly6G and DHR123) cells. (TIF) [file ppat.1006054.s002.tif]

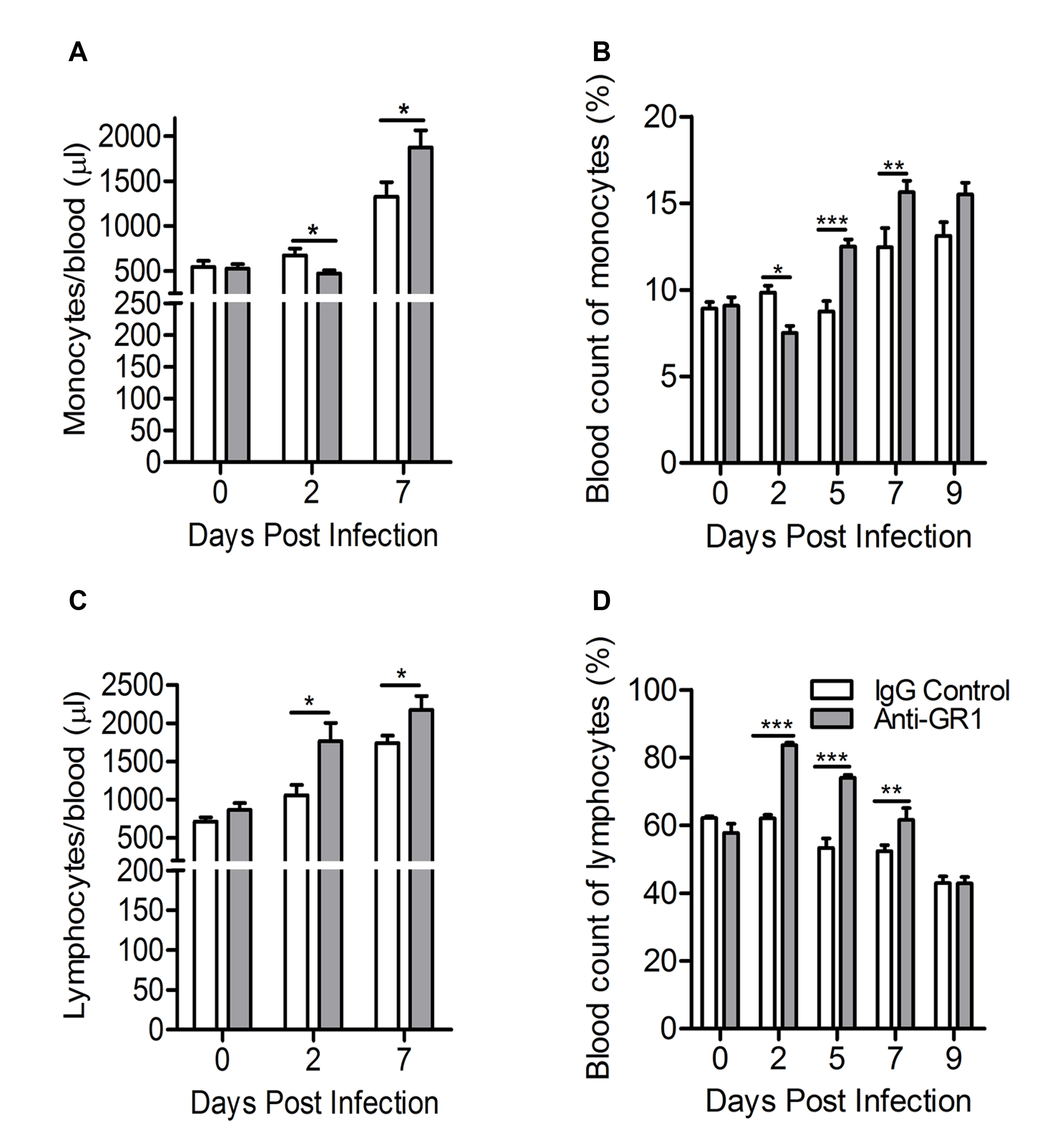

Supplement: S3 Fig — (A) Number of monocytes measured via flow cytometry and (B) blood smears from infected control (CTR) and anti-GR1-treated mice. (C) Number of lymphocytes measured via flow cytometry and (D) blood smears from control (CTR) and anti-GR1-treated mice. Data are representative of two independent experiments and are expressed as the mean ± SEM (Kruskal-Wallis test where * p <0.05, ** p <0.01 and *** p <0.001; n = 10–20 mice/experiment). (TIF) [file ppat.1006054.s003.tif]

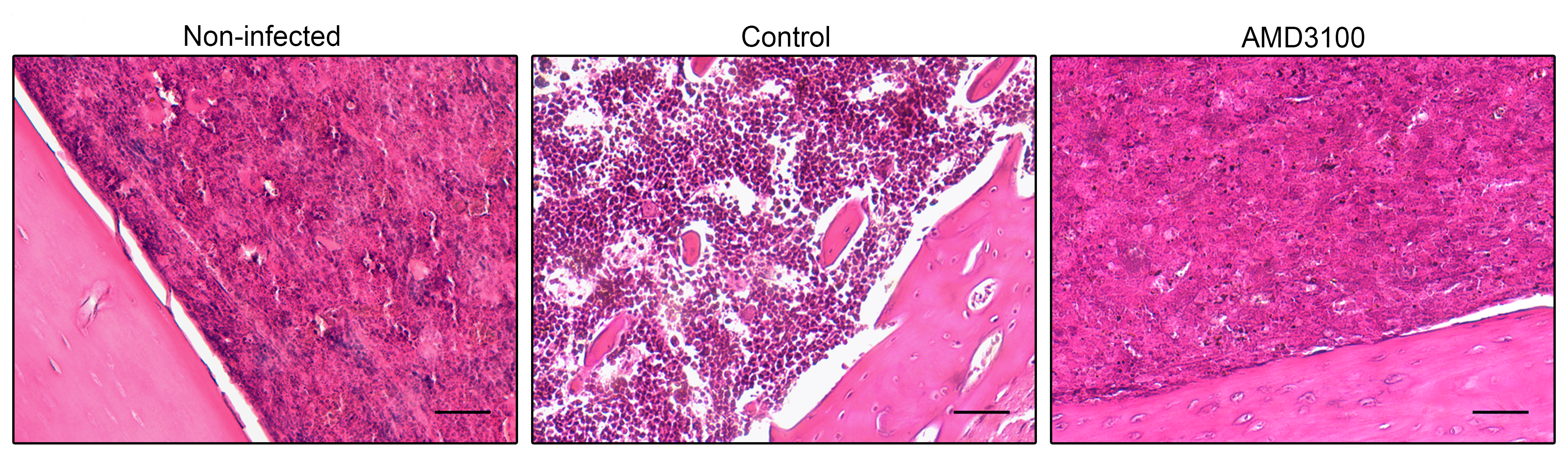

Supplement: S4 Fig — Bone marrow samples from non-infected mice, mice infected with P. berghei ANKA on the day of death (9th for ALI/ARDS) and infected and treated with AMD3100 mice (21st dpi). Note that the cellularity in mice infected and then treated is considerably higher than in infected, untreated mice (200x, scale bar 50 μm). (TIF) [file ppat.1006054.s004.tif]

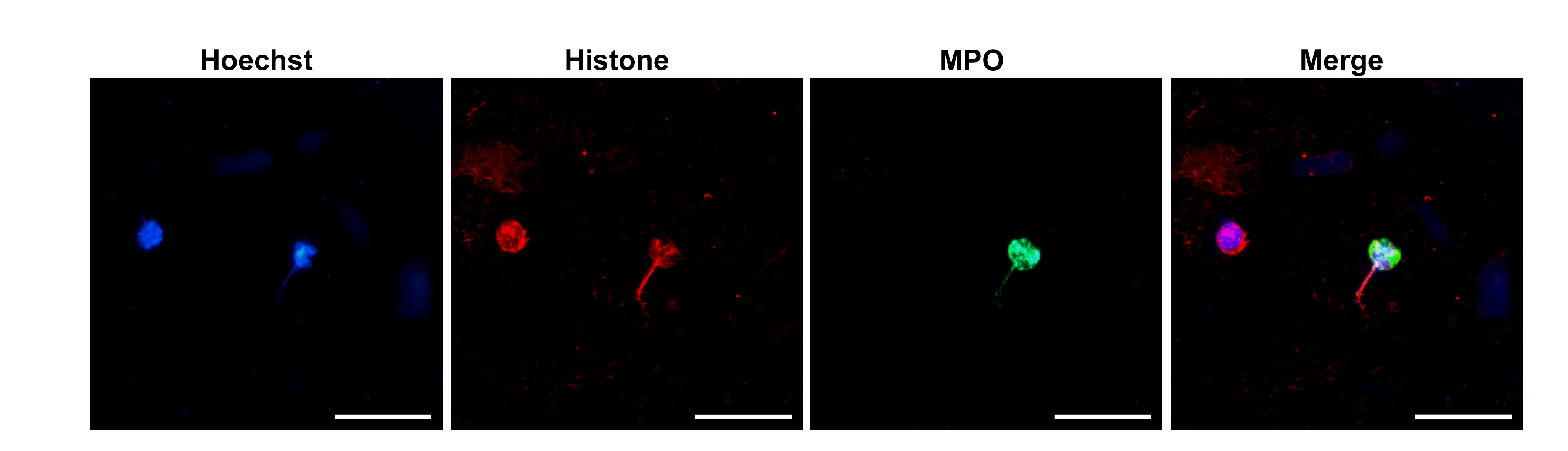

Supplement: S5 Fig — Smear of peripheral blood of PbA infected DBA2 mice stained for DNA (blue), histone (red), and myleporoxidase (green). Overlapping colors indicate filamentous structures (NETs). (1000x, scale bar 20 μm). (TIF) [file ppat.1006054.s005.tif]
